# Combination of ADAM17 knockdown with eplerenone is more effective than single therapy in ameliorating diabetic cardiomyopathy

**DOI:** 10.3389/fphar.2024.1364827

**Published:** 2024-05-10

**Authors:** Lin Xie, Dejin Zang, Jianmin Yang, Fei Xue, Wenhai Sui, Yun Zhang

**Affiliations:** ^1^ National Key Laboratory for Innovation and Transformation of Luobing Theory, The Key Laboratory of Cardiovascular Remodeling and Function Research, Chinese Ministry of Education, Chinese National Health Commission and Chinese Academy of Medical Sciences, Department of Cardiology, Qilu Hospital of Shandong University, Jinan, China; ^2^ Cardiovascular Disease Research Center of Shandong First Medical University, Central Hospital Affiliated to Shandong First Medical University, Jinan, China

**Keywords:** diabetic cardiomyopathy, ADAM17, eplerenone, RAAS, fibrosis

## Abstract

**Background:**

The renin-angiotensin-aldosterone system (RAAS) members, especially Ang II and aldosterone, play key roles in the pathogenesis of diabetic cardiomyopathy (DCM). Angiotensin-converting enzyme inhibitors or angiotensin-receptor blockers combined with aldosterone receptor antagonists (mineralocorticoid receptor antagonists) have substantially improved clinical outcomes in patients with DCM. However, the use of the combination has been limited due to its high risk of inducing hyperkalemia.

**Methods:**

Type 1 diabetes was induced in 8-week-old male C57BL/6J mice by intraperitoneal injection of streptozotocin at a dose of 55 mg/kg for 5 consecutive days. Adeno-associated virus 9-mediated short-hairpin RNA (shRNA) was used to knock down the expression of ADAM17 in mice hearts. Eplerenone was administered via gavage at 200 mg/kg daily for 4 weeks. Primary cardiac fibroblasts were exposed to high glucose (HG) *in vitro* for 24 h to examine the cardiac fibroblasts to myofibroblasts transformation (CMT).

**Results:**

Cardiac collagen deposition and CMT increased in diabetic mice, leading to cardiac fibrosis and dysfunction. In addition, ADAM17 expression and activity increased in the hearts of diabetic mice. ADAM17 inhibition and eplerenone treatment both improved diabetes-induced cardiac fibrosis, cardiac hypertrophy and cardiac dysfunction, ADAM17 deficiency combined with eplerenone further reduced the effects of cardiac fibrosis, cardiac hypertrophy and cardiac dysfunction compared with single therapy *in vivo*. High-glucose stimulation promotes CMT *in vitro* and leads to increased ADAM17 expression and activity. ADAM17 knockdown and eplerenone pretreatment can reduce the CMT of fibroblasts that is induced by high glucose levels by inhibiting TGFβ1/Smad3 activation; the combination of the two can further reduce CMT compared with single therapy *in vitro*.

**Conclusion:**

Our findings indicated that ADAM17 knockout could improve diabetes-induced cardiac dysfunction and remodeling through the inhibition of RAAS overactivation when combined with eplerenone treatment, which reduced TGF-β1/Smad3 pathway activation-mediated CMT. The combined intervention of ADAM17 deficiency and eplerenone therapy provided additional cardiac protection compared with a single therapy alone without disturbing potassium level. Therefore, the combination of ADAM17 inhibition and eplerenone is a potential therapeutic strategy for human DCM.

## Graphical Abstract

**Figure:**
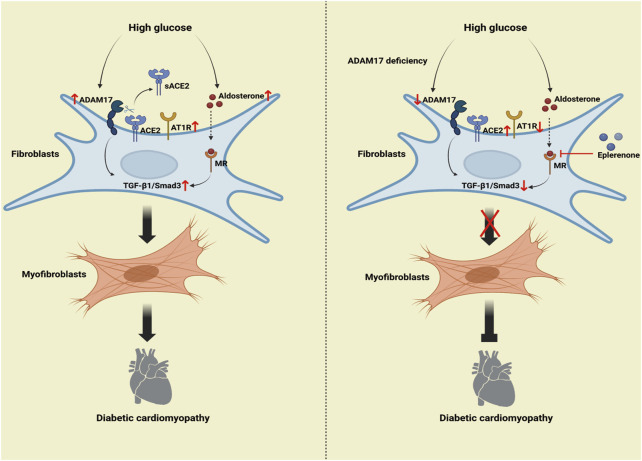
Mechanism of the roles of combination of ADAM17 knockdown and eplerenone treatment in DCM-induced cardiac fibrosis. The high-glucose activated RAAS increased ADAM17 activity, ACE2 shedding, and AT1R expression, thereby activating TGF-β1/Smad3 signaling in cardiac fibroblasts, which increased myofibroblast transformation and cardiac collagen deposition in DCM. The overactivated RAAS increased aldosterone expression, so aldosterone then bound to aldosterone receptors to further promote TGF-β1/Smad3 pathway-mediated myofibroblast transformation. ADAM17 deletion weakened ACE2 shedding and AT1R expression, inactivated TGF-β1/Smad3 signaling in myocardial fibroblasts, and resulted in reduced myofibroblast transformation and cardiac collagen deposition in DCM. Moreover, eplerenone acted as an aldosterone receptor antagonist to reduce the aldosterone activation of TGF-β1. Eplerenone combined with ADAM17 deficiency can further reduce the fibroblast transformation and collagen deposition caused by diabetes and improve the cardiac fibrosis and cardiac remodeling caused by DCM compared with when either of the two is used as monotherapy.

## Introduction

The prevalence of diabetes mellitus (DM) has been continually increasing in the past decade, imposing a heavy burden on global public health. Cardiovascular complications secondary to DM have become a major challenge in the field of cardiovascular medicine (Bugger and Abel, 2014). Diabetic cardiomyopathy (DCM) was initially described as a human pathophysiological condition in which heart failure occurred in the absence of coronary artery disease, hypertension, and valvular heart disease, which considerably contribute to the increased mortality of diabetes mellitus worldwide (Dillmann, 2019). Despite numerous studies on DCM, there are few effective therapies. In addition, the pathology and molecular mechanism of DCM are yet unclear, which hinders the development of effective therapeutic targets. Therefore, further studies on novel therapeutic approaches of DCM treatments are necessary.

DCM initially presents with cardiac hypertrophy, diastolic dysfunction and partially reduced systolic function which leading to clinical restrictions due to heart failure symptoms (Dannenberg et al., 2021). DCM is characterized by myocardial hypertrophy, fibrosis and inflammation, as well as cardiomyocyte death (Boudina and Abel, 2007). There is ample evidence that patients with DM exhibit significant myocardial fibrosis, but no evidence-based therapies have been shown to have a beneficial effect on cardiac fibrosis in patients with diabetes (Dannenberg et al., 2021). Mechanistically, there are several pathological changes associated with DM that leading to cardiac functional alterations, such as metabolic disorders, oxidative stress, inflammatory response, altered calcium signaling, and activation of the renin-angiotensin-aldosterone system (RAAS) play major roles. RAAS members, especially Ang II and aldosterone, play key roles in cardiac fibrosis in DCM (Cambier et al., 2018; Wu et al., 2018). The administration of angiotensin-converting enzyme inhibitors (ACEIs) and angiotensin-receptor blockers (ARBs), as well as aldosterone receptor antagonists (mineralocorticoid receptor antagonists, MRAs), have substantially improved clinical outcomes in patients with DCM, however, these classic treatment are inadequate in counteracting an overactivated RAAS in patients with DCM due to their inherent limitations (Agarwal et al., 2021; Adamo et al., 2022). Therefore, new approaches are urgently needed to counteract RAAS overactivation in patients with DCM.

Eplerenone, a selective aldosterone receptor antagonist (Pitt et al., 2023), has fewer side effects for it is more selective to aldosterone receptors than spironolactone (Agarwal et al., 2021). Moreover, the RAAS plays an important role in the development of diabetic cardiomyopathy (Collier and McDonald, 2012). In a hyperglycemic environment, the local RAAS is often directly overactivated (Dillmann, 2019), causing aldosterone accumulation, which promotes the Ang II activity and fibrosis in the diabetic myocardium by upregulating profibrotic and oxidative mediators (Pitt et al., 2003; Brown, 2013). Improvement in left ventricular hypertrophy and structural remodeling through the use of MRAs has been confirmed in clinical research (Maron and Leopold, 2010; Leung et al., 2013). Johansen *et al.* found that eplerenone suppresses interstitial fibrosis in the subcutaneous adipose tissue of patients with type 2 diabetes (Johansen et al., 2021). Mahajan *et al.* found that eplerenone improved hemodynamic and ventricular dysfunction in streptozotocin (STZ)-isoproterenol-challenged rats (Mahajan et al., 2018). Overall, the potential utility of eplerenone for DCM treatment has been demonstrated in both by clinical and basic research. However, the mechanisms underlying the cardioprotective effects of eplerenone on DCM remain unclear.

A disintegrin and metalloproteinase-17 (ADAM17), also known as tumor necrosis factor-alpha-converting enzyme, is a membrane-bound enzyme that proteolytically releases multiple cell surface proteins such as cytokines and cytokine receptors to regulate their bioavailability (Scheller et al., 2011). In recent years, ADAM17 has been implicated in many etiological factors and in playing an important role in organ interstitial fibrosis, including liver fibrosis (Cai et al., 2020), lung fibrosis (Liu et al., 2018), kidney fibrosis (Kefaloyianni et al., 2016), and vascular fibrosis (Takayanagi et al., 2016). Furthermore, ADAM17 silencing prevents Ang II-induced cardiac hypertrophy and fibrosis in mice (Wang et al., 2009). ADAM17 acts by regulating angiotensin-converting enzyme-2 (ACE2) shedding, as a member of the RAAS. ACE2 cleaves Ang II to produce angiotensin-(1–7) (Ang 1–7) and thus acts as a negative regulator of the RAAS (Simões e Silva and Teixeira, 2016). Our previous studies showed that ADAM17 increased cardiac fibrosis by regulating ACE2 shedding in diabetic mice (Cheng et al., 2022). ACE2 overexpression improved left ventricular remodeling and function in a rat model of DCM (Dong et al., 2012). Several ADAM17 inhibitors have been screened for cancer research, but the high homology between metalloproteinases active sites has limited the development of highly specific ADAM17 small molecule inhibitors, and the clinical use has been limited due to its potential side effects (Rossello et al., 2016).

Myocardial fibrosis, a pathological change in DCM, contributes to the development of myocardial remodeling (Jia et al., 2018). Approximately 60%–70% of the cells in the heart are cardiac fibroblasts, which normally remain stationary and secrete extracellular collagen, which maintains the structural integrity and normal function of the heart. In DCM, cardiac fibroblasts to myofibroblasts transformation (CMT) is the initial step in myocardial fibrosis (Zhou et al., 2018). Myofibroblasts highly express α-smooth muscle actin (α-SMA) (He et al., 2018). Fibroblast activation protein (FAP), a membrane-bound proline-specific serine protease, is not expressed in normal fibroblasts but is expressed in myofibroblasts, so is a specific marker of myofibroblasts (Goldsmith et al., 2014). In addition, the TGF-β1/Smad3 pathway regulated cardiac fibrosis in mice with myocardial infarction by regulating the CMT. Targeting CMT, which is regulated by TGF-β1, may offer new hope for preventing DCM.

In large-scale clinical trials, the addition of aldosterone receptor antagonists to standard medical therapies, including ACEIs or ARBs, has beneficial effects on the prognosis of patients experiencing heart failure (Nagatomo et al., 2014). However, this treatment increases the risk of hyperkalemia (Leung et al., 2013). ADAM17 knockout and eplerenone can be used to effectively treat DCM. However, whether combination therapy is more effective than single therapy and whether this combination therapy causes adverse side effects, such as hyperkalemia, remains unclear. To test this hypothesis, we investigated the mechanism through which ADAM17 knockout combined with eplerenone therapy ameliorates left ventricular remodeling and function in a mouse model of DCM *in vivo* and whether ADAM17 knockout combined with eplerenone treatment reduces CMT in rat cardiac fibroblasts *in vitro*.

## Materials and methods

### Ethics statement

All animal experimental protocols complied with the Animal Management Rules of the Chinese Ministry of Health (Document no. 55, 2001) and conformed to National Institutes of Health (NIH) guidelines (the Guide for the Care and Use of Laboratory Animals; NIH Publication No. 85-23, revised 1996). All mice were maintained under specific pathogen-free, environmentally controlled (Temperature: 20°C–25°C; humidity: 50% ± 5%) barrier conditions in individual ventilated cages and were fed with sterile food and water.

### Materials

Streptozotocin (STZ) was purchased from MCE (USA). Eplerenone was purchased from BOC Sciences (USA). Masson’s trichrome, Sirius red and hematoxylin and eosin (H&E) staining kits were purchased from Solarbio (Beijing, China). Fluorescein isothiocyanate-conjugated wheat germ agglutinin (FITC-conjugated WGA) staining was purchased from Sigma-Aldrich (USA). *In situ* cell death detection kit was purchased from Roche (USA). The SensoLyte 520 TACE (α-secretase) activity assay kit was purchased from AnaSpec, Fremont (CA). RNeasy mini kit was purchased from Qiagen (Germany). The primary antibodies used are listed in Supplementary Table S3 and the primer sequences were listed in Supplementary Table S4.

### Adeno-associated virus 9 mediated gene knockdown

A type 9 adeno-associated virus (AAV9) has been reported to be the most effective virus for cardiac genetic intervention (Pacak et al., 2006). To knock down ADAM17 in the myocardium in C57BL/6J male mice, a AAV9 serotype system carrying scramble shRNA (shNC) and ADAM17 shRNA (shA17) was constructed by Genechem Technologies (Shanghai, China). Each mouse was injected with 200 μL of AAV9-shNC or AAV9-shA17 at a titer of 5 × 10^11^ vg through tail vein.

### Animal model and grouping

**FIGURE 1:**
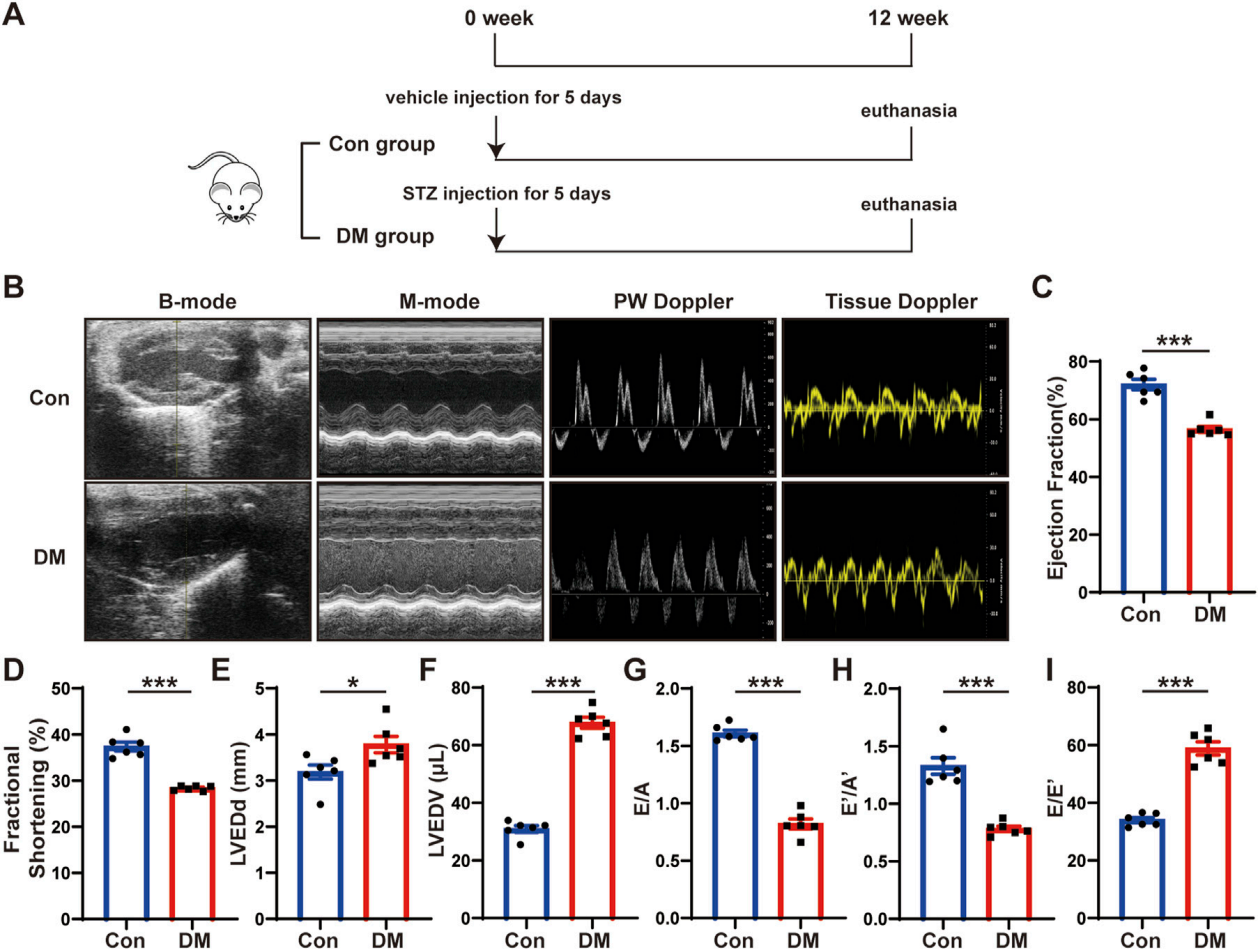
Time line of experimental studies *in vivo* and echocardiographic measurements of control and diabetic mice hearts. **(A)** Animal grouping and time line in first part of experimental studies *in vivo*. **(B)** Representative two-dimensional echocardiograms (first row), M-mode echocardiograms (second row), pulse-wave Doppler mode of mitral inflow (third row), and tissue Doppler mode of mitral annulus (fourth row). **(C)** Measurements of left ventricular ejection fraction (LVEF) in two groups of mice. **(D)** Measurements of fractional shortening (FS) in two groups of mice. **(E)** Measurements of left ventricular end-diastolic diameter (LVEDd) in two groups of mice. **(F)** Measurements of left ventricular end-diastolic volume (LVEDV) in two groups of mice. **(G)** Measurements of ratio of early to late left ventricular filling velocity (E/A) in two groups of mice. **(H)** Measurements of ratio of early to late diastolic peak annular velocity (E’/A’) in two groups of mice. **(I)** Measurements of the ratio of early diastolic transmittal inflow velocity to early diastolic mitral annulus velocity (E/E’) in two groups of mice. Values shown were mean and SEM (*n* = 6 in each group). *p < 0.05; ***p < 0.001.

The animal experiments were divided into two parts. In the first part of the *in vivo* experiments (Figure 1A), eight-week-old male C57BL/6J mice, purchased from Vital River Laboratory Animal Technology Co., Ltd. (Beijing, China), were used. After 1 week of adaptive feeding, the mice were randomly divided into two groups: a control group and a diabetes mellitus (DM) group. The mice in the DM group were injected intraperitoneally with STZ (MCE, USA) at a dosage of 55 mg/kg daily for five consecutive days, whereas the mice in the control group were injected with citrate buffer alone (Meng et al., 2023). Blood glucose levels were measured using an Accu-Check Active Glucometer (Roche, Shanghai, China). Mice with random glucose levels higher than 16.67 mmol/L were defined as having diabetes. A total of 12 weeks after STZ injection, mice hearts and serum were used for assays. In the second part of the *in vivo* experiments (Figure 3A), eight-week-old male C57BL/6J mice were injected with STZ according to the above-described method and subsequently randomly divided into four groups: DM + shNC + saline, DM + shA17 + saline, DM + shNC + Epl, and DM + shA17 + Epl group. AAV9-A17 knock-down and AAV9-NC viruses were injected to diabetic mice through the tail vein 12 weeks after STZ injection. Eplerenone, was administered at a dosage of 200 mg/kg per day for 1 month starting 2 weeks after AAV9 virus injection based on previous studies. These studies demonstrated that 200 mg/kg per day eplerenone effectively attenuated myocardial fibrosis in the angiotensin II induced hypertensive mouse and inhibited atrial fibrosis in mutant TGF-β1 transgenic mice (Nishioka et al., 2007; Chen XQ. et al., 2016). Consequently, our study employed 200 mg/kg per day dose of eplerenone to explore the effects on DCM. The DM + shNC + saline and DM + shA17 + saline mice were administered the same amount of normal saline. One month after eplerenone and saline administration, mice hearts and serum were collected for analysis. The experimental design detail of *in vivo* experiment in tabular form is listed in Supplementary Table S1.

### Body weight and biochemical assay

The body weight of the mice was measured by an electronic balance (Shimadzu Corp., Japan). Blood glucose of the mice was measured in tail venous blood by an Accu-Chek glucose meter with matched blood glucose strips (Roche, Germany). Serum lipid levels, including serum total cholesterol (TC) and serum triglyceride (TG), serum potassium concentrations and serum albumin (ALB) levels, blood urea nitrogen (BUN) and serum creatinine (Scr) were measured by an automatic biochemical analyzer (Chemray 240, Shenzhen, China).

### Blood pressure and heart rate measurement

At the end of the experiment, the blood pressure and heart rate of the mice were measured using a mouse tail-cuff blood pressure analysis system (Softron BP-98A, Japan). All the mice were first acclimated to the device to ensure accurate and reproducible measurements. The measurements were recorded between 9 a.m. and 12 p.m. in a warm and quiet environment by the same investigator. The mice were warmed inside a hyperthermia cylinder at a temperature of about 36°C–40°C for 5 min according to the manufacturer’s instructions, then the cuff sensor was placed at the base of the tail to record the measurements. At least three consecutive measurements were recorded to obtain the average values from each mouse.

### Echocardiography

Transthoracic echocardiography was performed using Visual Sonics Vevo 770 machine using a 30 MHz high frequency MS400 transducer (Visual Sonics, Canada). Anesthesia (5% isoflurane) was administered, and the mice remained under general anesthesia with continuous administration of 2% isoflurane during echocardiogram acquisition. Indices of systolic function were obtained from long-axis M-mode scans. The collected parameters included left ventricular ejection fraction (LVEF), fractional shortening (FS), left ventricular end-diastolic diameter (LVEDd), left ventricular end-diastolic volume (LVEDV), ratio of early to late diastolic mitral flow velocities (E/A), ratio of early to late diastolic mitral annular velocities (E’/A’), and ratio of early diastolic transmittal flow velocity to early diastolic mitral annular velocity (E/E’) were calculated.

### Histology and immunohistochemistry

The hearts of mice were isolated and fixed in 4% paraformaldehyde, embedded in paraffin, and cut into 5 μm thick sections for subsequent analyses. Masson’s trichrome, Sirius red and hematoxylin and eosin (H&E) staining were performed according to the manufacturer’s instructions using staining kits. Fluorescein isothiocyanate-conjugated wheat germ agglutinin (FITC-conjugated WGA) staining was used to measure the cardiomyocyte cross-sectional size. For immunohistochemical staining of tissue sections, the sections were dewaxed and subjected to antigen retrieval with citrate buffer (pH 6.0), followed by treatment with 3% H_2_O_2_. The sections were then blocked with 5% goat serum for 30 min at 37°C and incubated with primary antibodies at 4°C overnight. The next day, the sections were incubated with horseradish peroxidase (HRP)-conjugated secondary antibodies (ZSGB-Bio, Beijing, China) for 30 min at room temperature, and detection was performed using a 3′-diaminobenzidine (DAB) kit (ZSGB-Bio, Beijing, China). Hematoxylin was used for nuclear staining. Sections reacting with nonimmune immunoglobulin G (IgG) as well as secondary antibodies were used as negative controls. All histological images were examined and photographed under a microscope (Ti-S, Nikon) and analyzed with the Image-Pro Plus 6.0 software (Media Cybernetics Inc., USA). The primary antibodies used are listed in Supplementary Table S3.

### TdT-mediated dUTP nick end-labeling (TUNEL) staining

Apoptotic cells in the myocardium were detected via TUNEL assay, performed using a commercially available kit (*In Situ* Cell Death Detection Kit, TMR red) following the manufacturer’s instructions. Myocardial sections (5 μm thick) were permeabilized in PBS with 0.1% Triton X-100 and stained by TUNEL, subsequently sealed with DAPI tablet. The images were acquired with a fluorescence microscope (Ni-E, Nikon, Japan) with an excitation wavelength. The apoptosis ratio was expressed as the proportion of apoptotic cells to the total number of cells.

### Enzyme linked immunosorbent assay (ELISA)

Serum Hemoglobin A1c (HbA1c), IL-1β, IL-6, TNF-α, IL-4, and IL-10 levels in mice were measured using mouse HbA1c, IL-1β, IL-6, TNF-α, IL-4, and IL-10 ELISA kit following the manufacturer’s instructions (ANRK, China).

### Cell treatment

Primary cardiac fibroblasts were extracted (Meng et al., 2023) and cultured in Dulbecco’s modified Eagle’s medium (DMEM) containing 10% fetal bovine serum at 37°C under 5% CO2. To examine the effect of different concentrations of glucose on ADAM17 expression in cardiac fibroblasts, cardiac fibroblasts at 60% confluence were randomly divided into 6 groups, which were exposed to different treatments in the first part of the *in vitro* experiments (Figure 6A): (1) 5.5 mM glucose (low-glucose control, LG); (2) a combination of 5.5 mM glucose and 54.5 mM mannitol (high-osmotic-pressure control, HO); (3) 15 mM glucose; (4) 30 mM glucose; (5) 45 mM glucose; (6) 60 mM glucose. In the second part of the *in vitro* experiments (Figure 6B), cardiac fibroblasts at 60% confluence were randomly divided into 6 groups, which were exposed to different treatments: (1) 5.5 mM glucose (low glucose, LG); (2) 60 mM glucose (high glucose, HG); (3) NC-siRNA prior to high-glucose treatment (HG + siNC + saline); (4) ADAM17-siRNA prior to high-glucose treatment (HG + siA17+saline); (5) NC-siRNA and eplerenone prior to high-glucose treatment (HG + siNC + Epl); and (6) ADAM17-siRNA and eplerenone prior to high-glucose treatment (HG + siA17 + Epl). Eplerenone (10 μM) was added 1 h before stimulation. The experimental design detail of *in vitro* experiment in tabular form is listed in Supplementary Table S2.

### Small interfering RNA (siRNA)-mediated gene silencing

The siRNA of ADAM17 or negative control siRNA were obtained from keyybio, Shandong, China. Cardiac fibroblasts were cultured in antibiotic-free medium. The ADAM17-siRNA and NC-siRNA were delivered to the cardiac fibroblasts by Lipofectamine iMAX reagent (Invitrogen, Carlsbad, CA) according to the manufacturer’s protocol. After 24 h of transfection, the supernatant was replaced with fresh medium. ADAM17 siRNA sequence: 5′--3′: CGA​GTT​GAT​AGC​AAA​GAG​A.

### ADAM17 activity assay

After treatment as previously described, mouse heart tissue and primary cardiac fibroblasts assayed each sample for ADAM17 activity according to the protocol of the SensoLyte 520 TACE (α-secretase) activity assay kit.

### Western blotting analysis

Total protein was extracted from heart tissues using the Total Protein Extraction Kit (Invent Biotechnologies, Plymouth, MN, United States), and protein was extracted from cardiac fibroblasts using RIPA lysis buffer. Equal amounts of extracted protein samples were separated using 10% sodium dodecyl sulfate-polyacrylamide gel electrophoresis; the resolved protein bands were transferred to a polyvinylidene fluoride membrane (Millipore, MA, United States). After incubation in 5% bovine serum albumin for 1 h at room temperature, the membranes were incubated with primary antibodies at 4°C overnight. Following incubation with peroxidase-conjugated secondary antibodies (1:5000, Jackson ImmunoResearch Laboratories, PA, United States) at room temperature for 1 h, the protein bands were detected using a chemiluminescent substrate (Millipore, MA, United States) and exposure to a chemiluminescence instrument (GE, Amersham Imager 800RGB). The primary antibodies used are listed in Supplementary Table S3.

### Quantitative real-time polymerase chain reaction

Total RNA was extracted from isolated heart tissue by means of the RNeasy mini kit (Qiagen, 74704, Germany) which was reversed-transcribed using a PrimeScript RT reagent kit with gDNA Eraser (TaKaRa, Japan), and quantitative real-time RT-PCR was performed utilizing Takara SYBR RT-PCR kits according to the manufacturer’s instructions. Cycling conditions were: 95°C for 10 min, and 95°C for 15 s, 55°C for 15 s, and 72°C for 20 s for 40 cycles. Data were normalized by the level of *β-actin* expression in each individual sample, and the 2^−ΔΔCT^ method was used to calculate relative expression changes. The primer sequences were listed in Supplementary Table S4.

### Statistical analysis

All data were presented as mean ± SEM. All analyses were performed with GraphPad Prism 8 (GraphPad, CA). Normality assumption of the data distribution was assessed using Shapiro-Wilk test. For normally distribution, data were analyzed by unpaired two-tailed Student's t-tests to determine the statistical difference between two groups, and one-way ANOVA were performed to determine the statistical difference between multiple groups. In all statistical comparisons, *p*-values < 0.05 was considered to denote statistically significance. Non-significant *p*-values were not shown.

## Results

### Cardiac systolic and diastolic function impaired in DCM mice

To investigate the mechanisms of DCM, we established a mouse model of DCM as described in the first part of the *in vivo* experiment (Figure 1A). We intraperitoneally injected streptozotocin (STZ) into eight-week-old C57BL/6J male mice for five consecutive days. The blood glucose levels of mice were markedly elevated in the DM group compared with those in the control group and remained high until the end of the experiment. Twelve weeks after STZ injection, the mice hearts and serum were harvested. The blood glucose and hemoglobin A1c (HbA1c) levels of the mice in the DM group were substantially higher than those in the control group (Supplementary Figure S1A, B), whereas the body weight was considerably lower (Supplementary Figure S1C). The heart rate (Supplementary Figure S1D) and blood pressure (Supplementary Figures S1E–G) of the mice in the DM group did not substantially differ from those in the control group. However, the serum total cholesterol (Supplementary Figure S1H) and serum triglyceride (Supplementary Figure S1I) level were higher in the DM mice than in the control mice; the potassium concentration (Supplementary Figure S1J), albumin (ALB) (Supplementary Figure S1K), blood urea nitrogen (BUN) (Supplementary Figure S1L), and serum creatinine (Scr) levels (Supplementary Figure S1M) did not significantly differ between the groups. The echocardiography and statistical results showed that compared with the control group, the cardiac systolic function of the mice in the DM group was deteriorated, which mainly manifested as decreased left ventricular ejection fraction (LVEF) and fractional shortening (FS), and increased left ventricular end-diastolic diameter (LVEDd) and left ventricular end-diastolic volume (LVEDV) (Figures 1B–F). The cardiac diastolic function of the mice in the DM group was worse than that of the control group, which manifested as decreased ratio of early to late diastolic mitral flow velocities (E/A), ratio of early to late diastolic mitral annular velocities (E’/A’) and increased ratio of early diastolic transmittal flow velocity to early diastolic mitral annular velocity (E/E’) (Figures 1G–I). These results showed that the diabetic mice experienced abnormal lipid metabolism and impaired cardiac function.

### DM promoted cardiac remodeling and increased myocardial ADAM17 expression and activity

**FIGURE 2:**
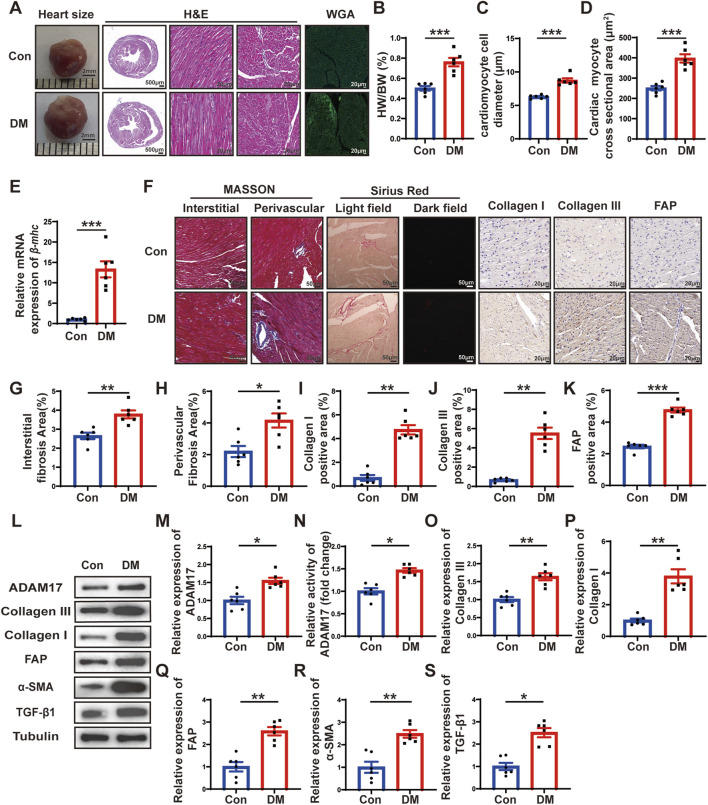
Histological staining and ADAM17 and cardiac fibrosis related molecular expression. **(A)** Representative images of heart sizes (scale bar = 2 mm), cardiac cross sections in hematoxylin and eosin (H&E) staining (scale bar = 500 μm), myocardial fiber tissue and cardiomyocyte cell diameter in H&E staining (scale bar = 20 μm), and cardiomyocyte cross-sectional areas in wheat germ agglutinin (WGA) staining (scale bar = 20 μm) in two groups of mice. **(B)** Measurements of heart weight/body weight (HW/BW) ratio in two groups of mice. **(C)** Quantification of the cardiomyocyte cell diameter in H&E staining in two groups of mice. **(D)** Quantification of cardiac myocyte cross-sectional area measured by WGA staining in two groups of mice. **(E)** Quantification of *β-mhc* mRNA expression in two groups of mice. **(F)** Representative Masson’s trichrome staining for myocardial interstitial and perivascular fibrosis (scale bar = 50 μm), representative Sirius Red staining for myocardial fibrosis (scale bar = 50 μm), representative Collagen I, Collagen III, and fibroblast activation protein (FAP) staining (scale bar = 50 μm) in two groups of mice. **(G,H)** Quantification of the interstitial and perivascular fibrosis area in two groups of mice. **(I–K)** Quantification of the Collagen I, Collagen III, and FAP expression in two groups of mice. **(L)** Representative Western blotting images of ADAM17, Collagen III, Collagen I, FAP, α-SMA and TGF-β1 expression in two groups of mice. **(M)** Quantification of ADAM17 expression in two groups of mice. **(N)** Quantification of ADAM17 activity in two groups of mice. **(O–S)** Quantification of Collagen III, Collagen I, FAP, α-SMA and TGF-β1 expression in two groups of mice. Values shown were mean and SEM (*n* = 6 in each group). *p < 0.05; **p < 0.01; ***p < 0.001.

To investigate the effects of DM on cardiac remodeling, heart size images were captured, and hematoxylin and eosin (H&E) staining and wheat germ agglutinin (WGA) staining were conducted. The results showed that the heart size, left ventricular cross-sectional area were larger in DM group than in the control group (Figure 2A). Moreover, the ratio of heart weight to body weight (HW/BW) of the mice in the DM group was markedly higher than that of the mice in the control group (Figure 2B). H&E staining showed that the diameter of cardiomyocytes in DM groups were significantly larger than that in control group (Figure 2C). WGA staining showed that cardiac myocyte cross sectional areas in DM group were significantly larger than that in control group (Figure 2D). In addition, qRT-PCR showed that *β-mhc* level in DM group was higher than that in the control group (Figure 2E). The results of Masson’s trichrome staining and Sirius Red staining showed that the left ventricular fibrotic area in the DM group was dramatically larger than that in the control group, and the expressions of Collagen I, Collagen III and FAP in the left ventricle of the DM group were higher than those in the control group, as identified through histochemical staining (Figures 2F–K). In addition, compared with the control group, the myocardial ADAM17 protein expression and activity level were higher in the DM group; the levels of fibrosis-related molecules collagen III and collagen I were higher than those in the control group (Figures 2L–P). Cardiac fibroblasts play an important role in the pathology of myocardial fibrosis by differentiating into myofibroblasts. FAP is the marker of CMT and α-SMA is a marker of myofibroblasts, whereas TGF-β1, is a factor promoting CMT. The Western blotting results showed that the expressions of FAP, α-SMA and TGF-β1 were increased in the DM group, which proved that CMT can be promoted by hyperglycemic environment, thereby accelerating diabetic myocardial fibrosis (Figures 2Q–S). These results suggested that sustained hyperglycemia promoted cardiac hypertrophy, myocardial collagen deposition and CMT, which promoted cardiac remodeling and increased the expression and activity of ADAM17 in the myocardium, suggesting that ADAM17 played a role in the occurrence and development of DCM.

### Combination of ADAM17 knockdown and eplerenone treatment further attenuated cardiac dysfunction compared with single therapy in DM mice

**FIGURE 3:**
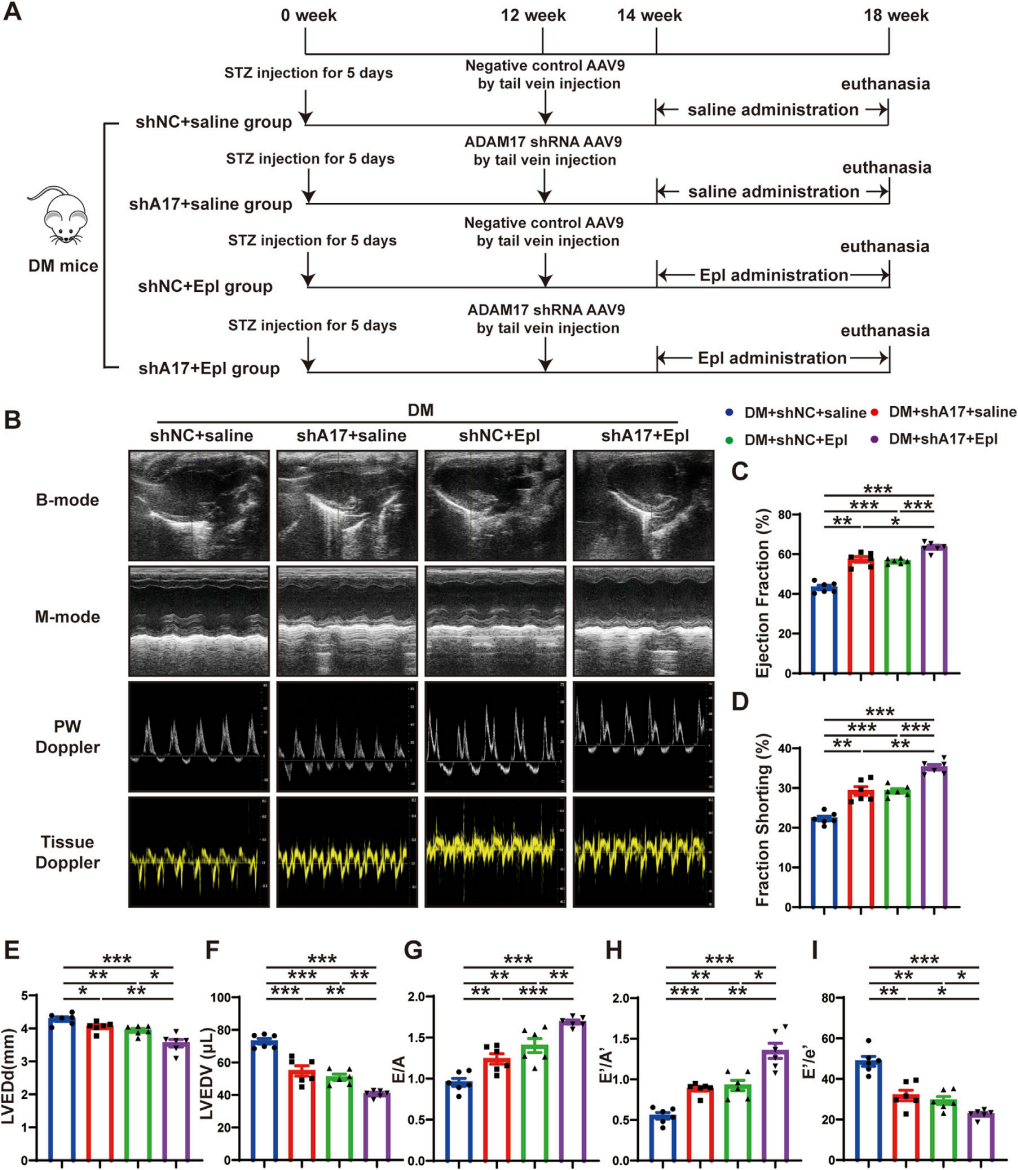
Time line of experimental studies *in vivo* and echocardiographic measurements in four groups of mice hearts. **(A)** Animal grouping and time line in second part of experimental studies *in vivo*. **(B)** Representative two-dimensional echocardiograms (first row), M-mode echocardiograms (second row), pulse-wave Doppler mode of mitral inflow (third row), and tissue Doppler mode of mitral annulus (fourth row). **(C)** Measurements of left ventricular ejection fraction (LVEF) in four groups of mice. **(D)** Measurements of fractional shortening (FS) in four groups of mice. **(E)** Measurements of left ventricular end-diastolic diameter (LVEDd) in four groups of mice. **(F)** Measurements of left ventricular end-diastolic volume (LVEDV) in four groups of mice. **(G)** Measurements of ratio of early to late left ventricular filling velocity (E/A) in four groups of mice. **(H)** Measurements of ratio of early to late diastolic peak annular velocity (E’/A’) in four groups of mice. **(I)** Measurements of the ratio of early diastolic transmittal inflow velocity to early diastolic mitral annulus velocity (E/E′) in four mice. Values shown were mean and SEM (*n* = 6 in each group). *p < 0.05; **p < 0.01; ***p < 0.001.

Because the expression and activity of ADAM17 in the myocardium of the DCM mice were elevated, and cardiac fibroblast differentiation is a salient feature of DCM, we investigated the effects of ADAM17 deficiency in cardiac fibroblasts and the use of the aldosterone receptor antagonist, eplerenone on the mice with DCM *in vivo*. The gene targeting ADAM17 is embryonic lethal (Liu et al., 2018). Our previous studies showed that mice with fibroblast conditional knockout of ADAM17 exhibit a strongly inflammatory state. Therefore, in this study, we used adeno-associated virus (AAV)9 to knock down ADAM17 expression and AAV9-shNC as a negative control, via tail vein injection. The results of the Western blotting analysis of the mouse heart tissues confirmed that ADAM17 protein expression was downregulated in AAV9-shA17 mice compared with that in the AAV9-shNC mice (Supplementary Figures S1N, O). Subsequently, the mice were administered eplerenone or saline, as described in the second part of the *in vivo* experiments (Figure 3A). The blood glucose level (Supplementary Figure S2A); HbA1c (Supplementary Figure S2B); body weight (Supplementary Figure S2C); heart rate (Supplementary Figure S2D); blood pressure (Supplementary Figures S2E–G); serum total cholesterol (Supplementary Figure S2H); serum triglyceride (Supplementary Figure S2I); serum potassium (Supplementary Figure S2J); serum albumin (ALB) (Supplementary Figure S2K); blood urea nitrogen (BUN) (Supplementary Figure S2L); and serum creatinine (Scr) levels (Supplementary Figure S2M) did not markedly differ among the four groups. At the end of the experiment, the echocardiography results showed that the LVEF and FS in the DM + shA17 + saline and DM + shNC + Epl groups were higher than those in the DM + shNC + saline group, whereas those in the DM + shA17 + Epl group were higher than those in the DM + shA17 + saline group and DM + shNC + Epl group (Figures 3B–D). In addition, the LVEDd and LVEDV in the DM + shA17 + saline and DM + shNC + Epl groups were lower than that in the DM + shNC + saline group, whereas that in the DM + shA17 + Epl group was lower than that in the DM + shA17 + saline and DM + shNC + Epl groups (Figures 3E, F). However, E/A and E’/A’ were higher in the DM + shA17+saline and DM + shNC + Epl groups than in DM + shNC + saline group, but the E/E' was lower in the former than in the latter. The E/A and E’/A’ levels in DM + shA17+Epl group were higher than those in DM + shA17 + saline and DM + shNC + Epl groups, whereas E/E’ level was lower in DM + shA17 + Epl group than in those two groups (Figures 3G–I). These results demonstrated that ADAM17 knockdown in combination with eplerenone treatment can further improve the systolic and diastolic dysfunction of mice with DCM without affecting blood pressure and disturbing blood potassium levels.

### Combination of ADAM17 knockdown and eplerenone treatment further attenuated cardiac remodeling compared with single therapy in DM mice

**FIGURE 4:**
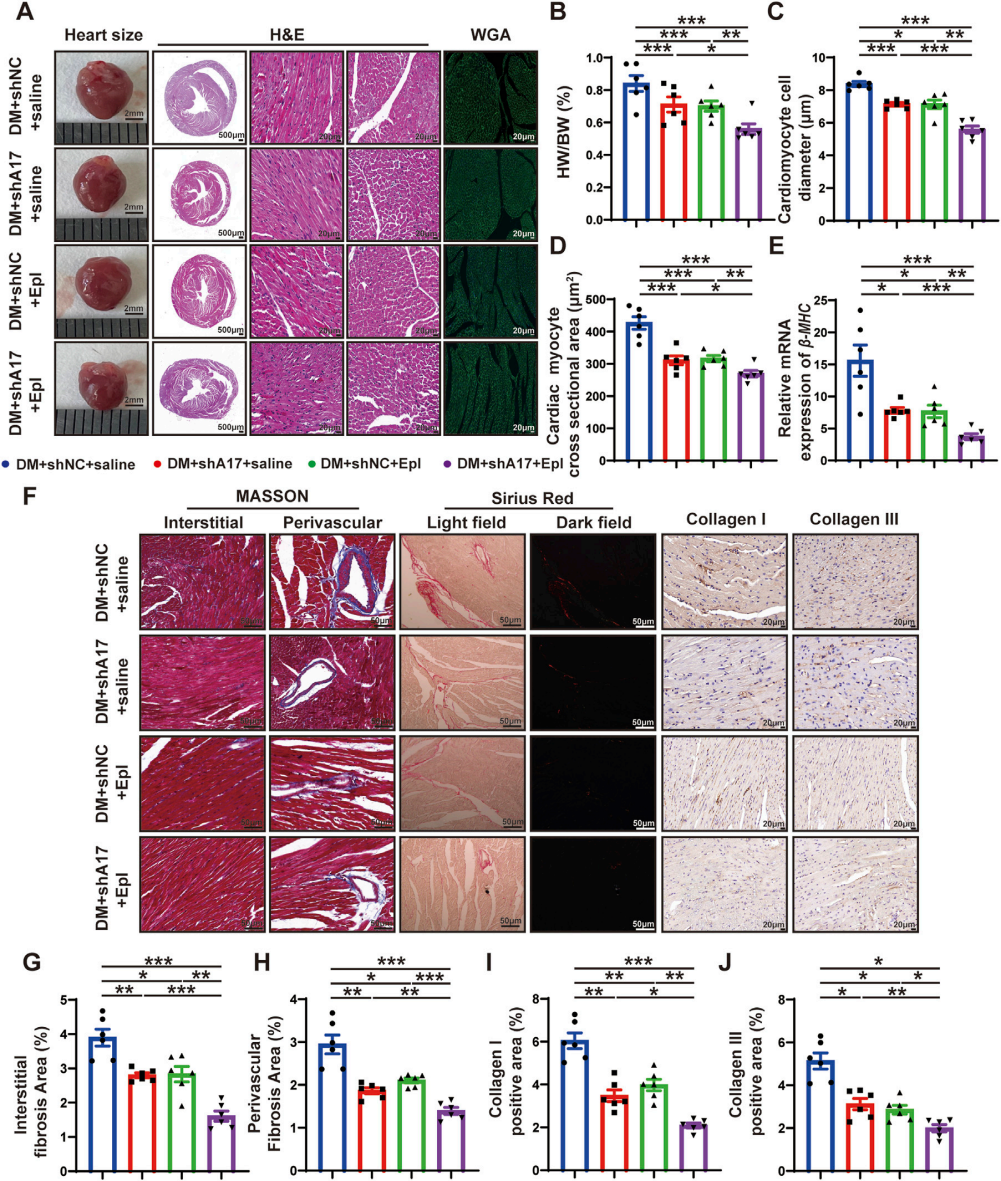
Histological staining in four groups of mice hearts. **(A)** Representative images of heart sizes (scale bar = 2 mm), cardiac cross sections in hematoxylin and eosin (H&E) staining (scale bar = 500 μm), myocardial fiber tissue and cardiomyocyte cell diameter in H&E staining (scale bar = 20 μm), and cardiomyocyte cross-sectional areas in wheat germ agglutinin (WGA) staining (scale bar = 20 μm) in four groups of mice. **(B)** Measurements of heart weight/body weight (HW/BW) ratio in four groups of mice. **(C)** Quantification of the cardiomyocyte cell diameter in H&E staining in four groups of mice. **(D)** Quantification of cardiac myocyte cross-sectional area measured by WGA staining in four groups of mice. **(E)** Quantification of *β-mhc* mRNA expression in four groups of mice. **(F)** Representative Masson’s trichrome staining for myocardial interstitial and perivascular fibrosis (scale bar = 50 μm), representative Sirius Red staining for myocardial fibrosis (scale bar = 50 μm), representative Collagen I and Collagen III staining (scale bar = 50 μm) in four groups of mice. **(G,H)** Quantification of the interstitial and perivascular fibrosis area in four groups of mice. **(I,J)** Quantification of the Collagen I and Collagen III expression in four groups of mice. Values shown were mean and SEM (*n* = 6 in each group). *p < 0.05; **p < 0.01; ***p < 0.001.

To investigate the effects of ADAM17 knockdown and eplerenone on cardiac remodeling caused by DCM, heart size images were obtained, and H&E staining was conducted. The results showed that the heart size and cardiac cross-sectional area of the mice in the DM + shA17 + saline and DM + shNC + Epl groups were lower than those of the mice in the DM + shNC + saline group. However, the heart size and cardiac cross-sectional area of the mice in the DM + shA17 + Epl group were further decreased compared with those of the mice in the DM + shA17 + saline and DM + shNC + Epl groups (Figure 4A). Moreover, compared with the DM + shNC + saline group, the HW/BW of the mice in the DM + shA17 + saline and DM + shNC + Epl groups were substantially lower; the levels were further decreased in the mice in the DM + shA17 + Epl group (Figure 4B).

H&E staining and WGA staining showed that the diameter of cardiomyocytes and cardiac myocyte cross sectional areas in the DM + shA17 + saline and DM + shNC + Epl groups were lower than those of the mice in the DM + shNC + saline group; the levels were further decreased in the mice in the DM + shA17 + Epl group (Figures 4C, D). In addition, qRT-PCR showed that the elevated *β-mhc* level in DM + shNC + saline group decreased in DM + shA17 + saline group and DM + shNC + Epl group, and further decreased in DM + shA17 + Epl group (Figure 4E). The results of Masson’s trichrome and Sirius Red staining showed that the left ventricular fibrosis areas of the mice in the DM + shA17 + saline and DM + shNC + Epl groups were remarkably lower than those of the mice in the DM + shNC + saline group, whereas the left ventricular fibrosis induced by diabetes was further improved in the DM + shA17 + Epl group compared with that in the above two groups. The expression levels of collagen I and collagen III were also consistent with the fibrotic changes shown by Masson’s trichrome and Sirius Red staining (Figures 4F–J). These results showed that cardiac hypertrophy and myocardial collagen deposition were considerably reduced by the combination of ADAM17 knockdown and eplerenone treatment compared with single therapy with ADAM17 deficiency or eplerenone administration.

### Combination of ADAM17 knockdown and eplerenone treatment further reduced CMT compared with single therapy in DM mice

**FIGURE 5:**
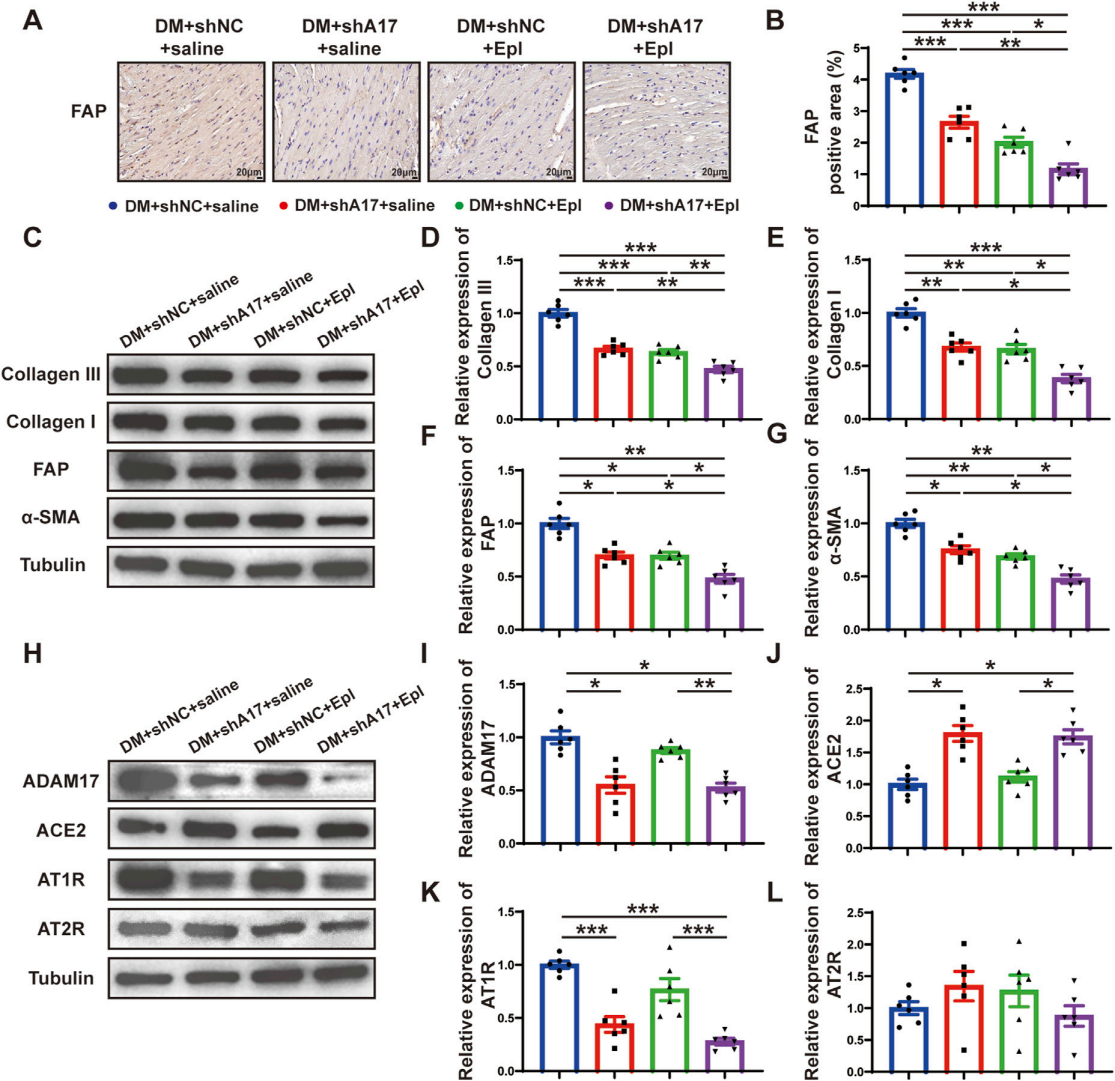
Cardiac fibroblasts transformation and RAAS activation following ADAM17 knockdown, eplerenone administration and combined treatment in DCM mice. **(A)** Representative of fibroblast activation protein (FAP) staining (scale bar = 20 μm) in four groups of mice. **(B)** Quantification of FAP expression in four groups of mice. **(C)** Representative Western blotting images of Collagen III, Collagen I, FAP and α-SMA expression in four groups of mice. **(D–G)** Quantification of Collagen III, Collagen I, FAP and α-SMA expression in four groups of mice. **(H)** Representative Western blotting images of ADAM17, ACE2, AT1R and AT2R expression in four groups of mice. **(I–L)** Quantification of ADAM17, ACE2, AT1R and AT2R expression in four groups of mice. Values shown were mean and SEM (*n* = 6 in each group). *p < 0.05; **p < 0.01; ***p < 0.001.

We examined the effects of ADAM17 knockdown and eplerenone treatment on CMT and cardiac fibrosis in mice with DCM. The results of histochemical staining showed that the expression of FAP in the DM + shA17 + saline and DM + shNC + Epl groups were lower than that in the DM + shNC + saline group, whereas the expression of FAP in the DM + shA17 + Epl group was further decreased compared with that in the DM + shA17 + saline and DM + shNC + Epl groups (Figures 5A, B). The Western blotting results showed that the expressions of collagen III, collagen I, FAP, and α-SMA in DM + shA17 + saline and DM + shNC + Epl groups were downregulated compared with those in the DM + shNC + saline group, whereas those in DM + shA17+Epl group were further downregulated compared with those in DM + shA17+saline and DM + shNC + Epl groups (Figures 5C–G). Furthermore, the Western blotting results showed that ADAM17 and angiotensin type 1 receptor (AT1R) expression were decreased in the DM + shA17 + saline group compared with those in the DM + shNC + saline group; those in the DM + shNC + Epl group were not substantially different. Compared with those in the DM + shA17 + saline group, the expression of ADAM17 and AT1R in the DM + shA17 + Epl group did no substantially change, whereas the expressions of ADAM17 and AT1R in the DM + shA17 + Epl group were markedly decreased compared with those in the DM + shNC + Epl group. Compared with the DM + shNC + saline group, protein expression level of ACE2 was higher in the DM + shA17 + saline and DM + shA17 + Epl groups, whereas the level was not notably different in the DM + shNC + Epl group. However, we observed no substantial difference in the angiotensin type 2 receptor (AT2R) levels among the four groups (Figures 5H–L). These results suggested that ADAM17 knockdown reduced CMT and affected cardiac fibrosis by inhibiting the activation of the RAAS. Eplerenone also reduced CMT and improved the effects of cardiac fibrosis. Moreover, the combination of ADAM17 knockdown and eplerenone treatment can further reduce CMT and improve the effects of cardiac fibrosis compared with single therapy alone.

### High-glucose treatment promoted CMT and increased ADAM17 expression and activity in cardiac fibroblasts *in vitro*

**FIGURE 6:**
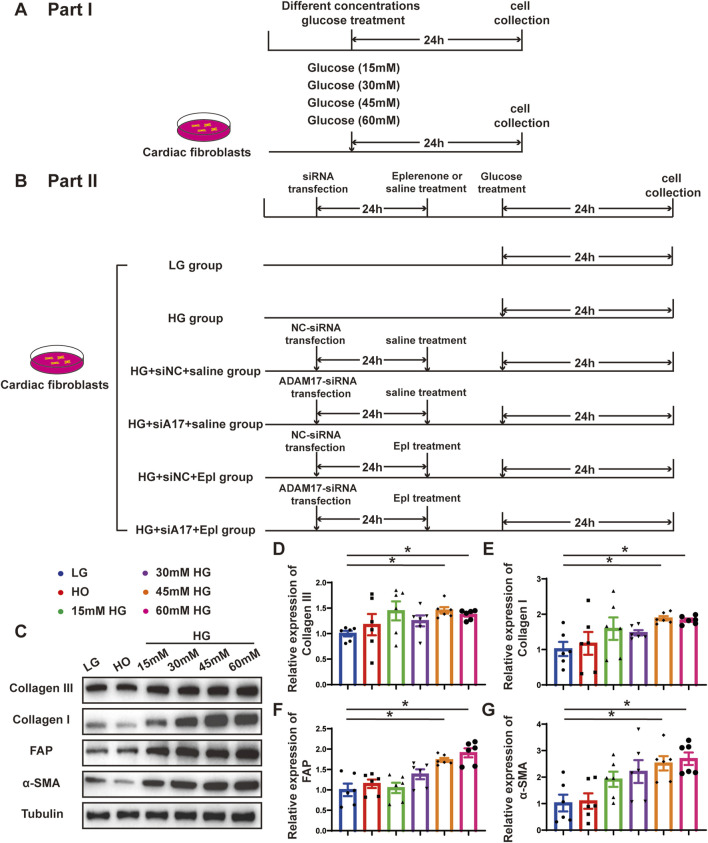
Time line of experimental studies *in vitro* and effect of glucose on primary cardiac fibroblasts transformation. **(A)** Cell treatment and time line in first part of experimental studies *in vitro*. **(B)** Cell treatment and time line in second part of experimental studies *in vitro.* **(C)** Representative Western blotting images of Collagen III, Collagen I, FAP and α-SMA expression in cardiac fibroblasts treated with different concentrations glucose. **(D–G)** Quantification of Collagen III, Collagen I, FAP and α-SMA expression in cardiac fibroblasts treated with different concentrations glucose. Values shown were mean and SEM (*n* = 6 in each group). *p < 0.05.

**FIGURE 7:**
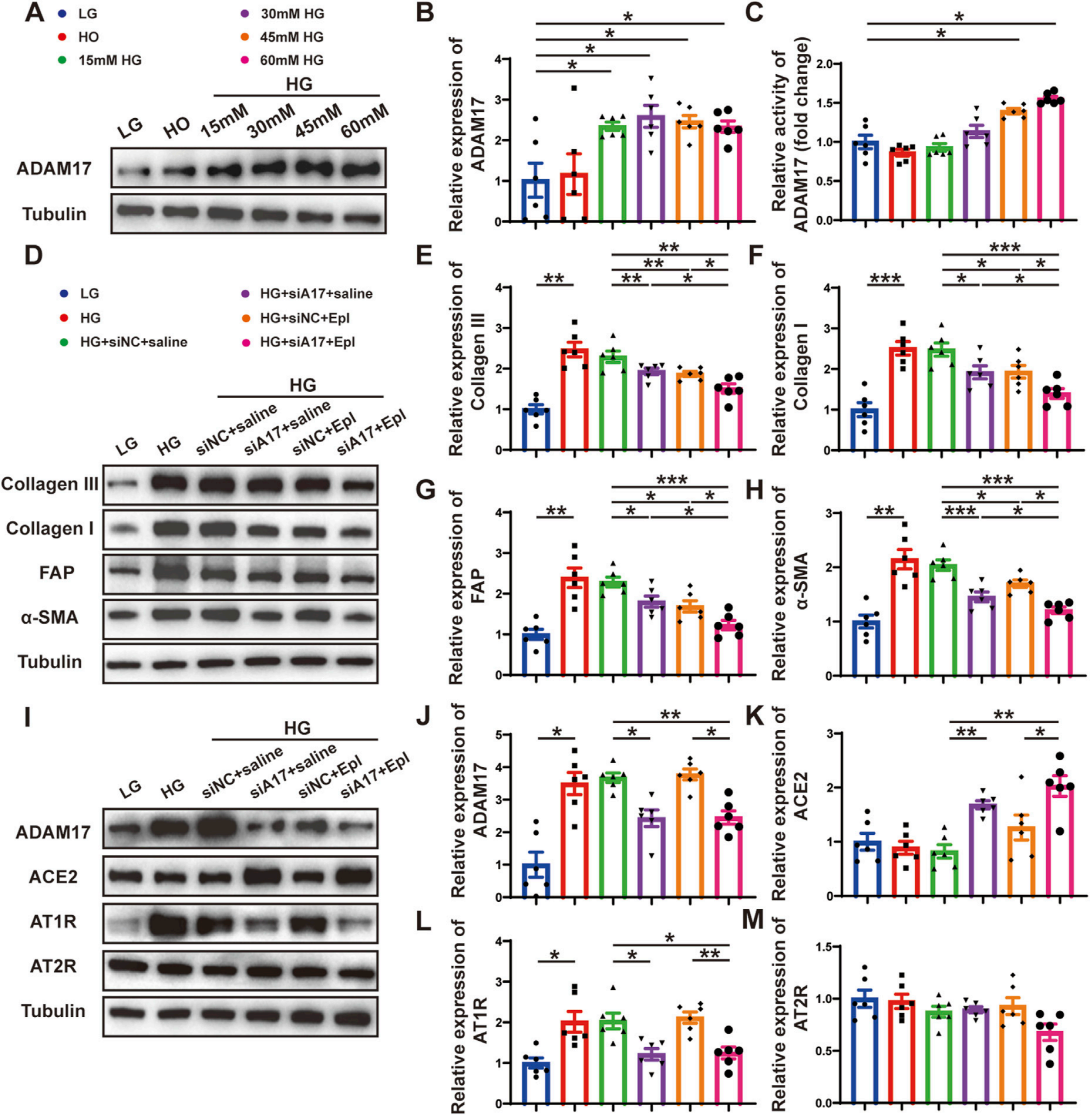
Cardiac fibroblasts transformation and RAAS activation following ADAM17 knockdown, eplerenone intervention and combined-administration in primary cardiac fibroblasts. **(A)** Representative Western blotting images of ADAM17 expression in cardiac fibroblasts treated with different concentrations glucose. **(B,C)** Quantification analysis of ADAM17 protein expression and activity in cardiac fibroblasts treated with different concentrations glucose. **(D)** Representative Western blotting images of Collagen III, Collagen I, FAP and α-SMA expression in cardiac fibroblasts treated with LG, HG, HG + siNC + saline, HG + siA17+saline, HG + siNC + Epl, HG + siA17+Epl, respectively. **(E–H)** Quantification of Collagen III, Collagen I, FAP and α-SMA expression in cardiac fibroblasts treated with LG, HG, HG + siNC + saline, HG + siA17+saline, HG + siNC + Epl, HG + siA17+Epl, respectively. **(I)** Representative Western blotting images of ADAM17, ACE2, AT1R and AT2R expression in cardiac fibroblasts treated with LG, HG, HG + siNC + saline, HG + siA17+saline, HG + siNC + Epl, HG + siA17+Epl, respectively. **(J–M)** Quantification of ADAM17, ACE2, AT1R and AT2R expression in cardiac fibroblasts treated with LG, HG, HG + siNC + saline, HG + siA17+saline, HG + siNC + Epl, HG + siA17+Epl, respectively. Values shown were mean and SEM (*n* = 6 in each group). *p < 0.05; **p < 0.01; ***p < 0.001.

To verify the effect of glucose on cardiac fibroblasts *in vitro*, we stimulated primary cardiac fibroblasts with different concentrations of glucose as described in the first part of the *in vitro* experiment (Figure 6A). The Western blotting results showed that the expressions of collagen III, collagen I, FAP, and α-SMA in cardiac fibroblasts gradually increased with the increase in glucose concentration (Figures 6C–G). Additionally, ADAM17 protein expression and activity in cardiac fibroblasts increased in a glucose-concentration- dependent manner (Figures 7A–C). These results suggested that high glucose levels promoted CMT *in vitro* and upregulated the expression and activity of ADAM17 in cardiac fibroblasts.

### Combination of ADAM17 knockdown and eplerenone treatment further reduced CMT compared with single therapy in cardiac fibroblasts *in vitro*

To further demonstrate the role of ADAM17 and eplerenone in CMT *in vitro*, we treated primary cardiac fibroblasts as described in the second part of the *in vitro* experiment (Figure 6B). The results of Western blotting showed that the expressions of collagen III, collagen I, FAP, and α-SMA were lower in the HG + siA17+saline and HG + siNC + Epl groups compared with HG + siNC + saline group. However, the expressions in the HG + siA17 + Epl group were event lower than those in the HG + siA17 + saline and HG + siNC + Epl groups (Figures 7D–H). Furthermore, the Western blotting results showed that ADAM17 and AT1R expressions were lower in the HG + siA17 + saline group than in the HG + siNC + saline group, but no notably different in the HG + siNC + Epl group. Compared with those in the HG + siA17 + saline group, the expression of ADAM17 and AT1R in the HG + siA17 + Epl group were not substantially different, whereas the expression of ADAM17 and AT1R in the HG + siA17 + Epl group were significantly lower than those in the HG + siNC + Epl group. Compared with the HG + siNC + saline group, the protein expression levels of ACE2 were higher in the HG + siA17+saline group and HG + siA17 + Epl group, but no different in the HG + siNC + Epl group. However, no substantial difference was observed in AT2R expression among the six groups (Figures 7I–M). These results suggested that ADAM17 knockdown and eplerenone treatment inhibited CMT by inhibiting RAAS activation and ADAM17 knockdown combined with eplerenone further inhibited CMT *in vitro*.

### Combination of ADAM17 knockdown and eplerenone treatment affected CMT via regulating TGF-β1/Smad3 pathway *in vivo* and *in vitro*

**FIGURE 8:**
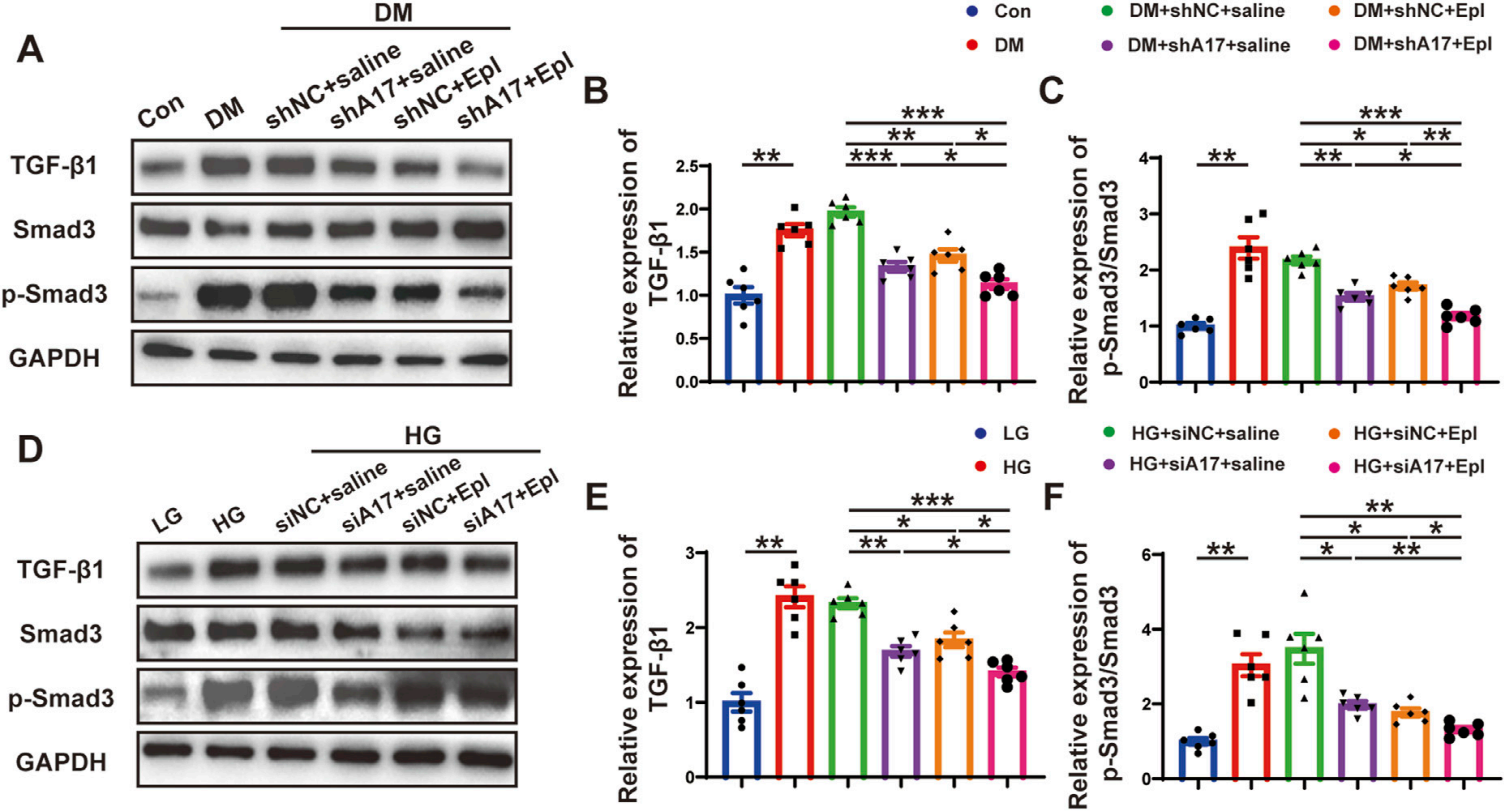
The activation of TGF-β1/Smad3 pathway and mechanism of the roles of combination of ADAM17 knockdown and eplerenone treatment in DCM-induced cardiac fibrosis. **(A)** Representative Western blotting images of TGF-β1, Smad3 and phospho-Smad3 expression in six groups of mice. **(B,C)** Quantification of TGF-β1 and the ratio of pSmad3/Smad3 in six groups of mice. **(D)** Representative Western blotting images of TGF-β1, Smad3 and phospho-Smad3 expression in cardiac fibroblasts treated with LG, HG, HG + siNC + saline, HG + siA17+saline, HG + siNC + Epl, HG + siA17+Epl, respectively. **(E,F)** Quantification of TGF-β1 and the ratio of pSmad3/Smad3 in cardiac fibroblasts treated with LG, HG, HG + siNC + saline, HG + siA17+saline, HG + siNC + Epl, HG + siA17+Epl, respectively. Values shown were mean and SEM (*n* = 6 in each group). *p < 0.05; **p < 0.01; ***p < 0.001.

We further examined the mechanisms underlying the effects of the combination of ADAM17 deficiency and eplerenone treatment in regulating CMT. TGF-β1 is a key profibrotic cytokine in DCM, and the TGF-β1/Smad3 pathway is a classic fibrosis pathway. Therefore, we examined the expression of phosphorylated Smad3 in the hearts of diabetic mice and found that phosphorylated Smad3 expression increased; however, ADAM17 knockdown and eplerenone treatment both decreased the expression of phosphorylation of Smad3. The combination of ADAM17 knockdown and eplerenone treatment further reduced the expressions of TGF-β1 and phosphorylated Smad3 *in vivo* (Figures 8A–C). Similarly, we found that high-glucose stimulation increased the expressions of TGF-β1 and phosphorylated Smad3 in cardiac fibroblasts *in vitro*, whereas ADAM17 deletion and eplerenone treatment decreased the expressions of TGF-β1 and phosphorylated Smad3 in cardiac fibroblasts. Consistent with the results of the *in vivo* experiments, ADAM17 knockdown combined with eplerenone treatment further reduced TGF-β1 and Smad3 phosphorylation in cardiac fibroblasts (Figures 8D–F). These results suggested that ADAM17 knockdown combined with eplerenone treatment inhibited fibroblast transformation by inhibiting TGF-β1/Smad3 pathway activation, thereby reducing cardiac fibrosis and improving cardiac remodeling in DCM.

## Discussion

The main finding of our study was that the combination of ADAM17 knockdown and eplerenone treatment can protect the heart by reversing left ventricular remodeling and dysfunction without changing blood pressure, blood glucose, or potassium levels in DCM mice. The underlying mechanism is that the overactivated RAAS is inhibited, which reduce the transformation of fibroblasts into myofibroblasts, mediated by the activated TGF-β1/Smad3 pathway, thereby leading to reducing the collagen synthesis and improving the cardiac remodeling induced by diabetes. In addition, the combination of ADAM17 inhibition and eplerenone administration reduced cardiac apoptosis and cardiac inflammation in diabetic mice, further improving diabetes-induced cardiac dysfunction (Supplementary Figure S3). The combined intervention including ADAM17 knockdown and eplerenone treatment provided an additional cardioprotective effect compared with that produced by monotherapy and produced no side effects, such as hyperkalemia, which was a crucial finding in the search for new treatments for DCM.

The primary pathological outcomes of DCM include cardiac hypertrophy, myocardial fibrosis, and extracellular collagen deposition. The activation of the RAAS plays vital role in the production and degradation of collagen (Agarwal et al., 2021), such as Ang II, which plays a key role in promoting myocardial fibrosis (Weber and Brilla, 1991). The renin-angiotensin system is the main regulator of aldosterone production, and Ang II acts through angiotensin receptors to stimulate aldosterone release. Aldosterone levels are higher in diabetic patients than in normal individuals, and the activation of aldosterone can lead to myocardial fibrosis. Therefore, the pharmacological disruption of aldosterone action at the tissue level may be particularly useful for patients with diabetes. Aldosterone binds to mineralocorticoid receptor (MR) to promote myocardial fibrosis; the specific mechanism may involve oxidative stress, inflammation, and apoptosis. MR is expressed in many tissues and cells including the kidney, heart, immune cells, and fibroblasts (Agarwal et al., 2021). The pathophysiological overactivation of MR leads to inflammation and fibrosis in cardiorenal disease. Cardiomyocyte-specific MR knockout also reduced inflammation and fibrosis in a mouse model of deoxycorticosterone acetate (DOCA)-salt-induced myocardial fibrosis (Fraccarollo et al., 2011). By blocking MR, eplerenone may attenuate cardiac steatosis and apoptosis and the subsequent remodeling and diastolic dysfunction in obese/type-II diabetic rats (Ramirez et al., 2013). Eplerenone, a selective aldosterone receptor antagonist, has been used in clinical studies to reduce morbidity and mortality in patients with acute myocardial infarction complicated by left ventricular dysfunction and heart failure (Pitt et al., 2023). In addition, a randomized controlled studies have shown that eplerenone reduces NT-proBNP levels in patients with worsening chronic heart failure and diabetes mellitus and/or chronic kidney disease (Filippatos et al., 2016).

Excessive RAAS activation leads to the excessive production of Ang II and aldosterone, playing an indispensable role in the pathogenesis of DCM. Aldosterone receptor antagonists are generally used in addition to administering ACEIs and ARBs to achieve the strongest therapeutic effect in diabetic patients with heart failure. Although aldosterone receptor antagonists in combination with ACEIs or ARBs can substantially improve cardiac remodeling and reduce adverse cardiovascular events in patients with heart failure; however, many limitations face their use. Firstly, ACEIs or ARBs in combination with aldosterone receptor antagonists may cause hyperkalemia and renal dysfunction to overrule the beneficial effects of this approach. In a randomized controlled trial designed to study the safety and efficacy of eplerenone in patients at a high risk of hyperkalemia and worsening renal function, eplerenone was effective in reducing the primary composite endpoint in all subgroups; however, the risks of hospitalization for hyperkalemia and discontinuation of investigational medication due to adverse events were increased (Eschalier et al., 2013). Secondly, although ACEIs inhibits Ang II production and ARBs inhibits Ang II binding to angiotensin receptors to inhibit the production of aldosterone, the so-called Ang II/aldosterone escape that often occurs for ACEIs cannot block the chymase-catalyzed Ang II, which usually produces more Ang II than the ACE process (Pitt et al., 1999). Finally, ARBs only block the binding of Ang II to AT1R on the cell membrane, whereas the Ang II synthesized in the cell cannot be blocked, while intracellular Ang II still caused myocardial fibrosis.

Therefore, a treatment that may inhibit Ang II production without disturbing blood potassium concentration when combined with aldosterone receptor antagonists is urgently needed. Previous studies in our laboratory showed that ACE2/Ang1-7/MasR is a novel axis for the treatment of cardiovascular diseases. ACE2 cleaves Ang II to produce Ang-(1–7) and thus acts as a negative regulator of RAS (Simões e Silva and Teixeira, 2016). Overexpression of ACE2 improved left ventricular remodeling and function in DCM rats (Dong et al., 2012). As a transmembrane protein, ADAM17 cleaves ACE2 on the cell surface via proteolysis, causing ACE2 to fall off and lose its protective effects on the myocardium. *Wang X et al.* found that ADAM17 silencing can strongly inhibit the myocardial hypertrophy and myocardial fibrosis induced by Ang II stimulation in spontaneously hypertensive and hypertensive rats; however, the specific molecular mechanism of this effect is unknown (Wang et al., 2009). ADAM17 knockdown attenuated whereas ADAM17 overexpression aggravated cardiac fibrosis by regulating ACE2 shedding in diabetic mice (Cheng et al., 2022). However, Ang II can activate ADAM17 and increase ACE2 shedding, forming positive feedback in the RAAS; that is, Ang II stimulation inhibits the degradation of Ang II by ACE2, thereby reducing myocardial protection of ACE2 (Patel et al., 2014). In this study, ADAM17 knockdown reduced the shedding of ACE2, thereby increasing ACE2 expression in a high-glucose environment, which had an antagonistic effect on Ang II and improved cardiac function in DCM. In addition, the combined administration of eplerenone in mice with DCM further improved cardiac remodeling without causing potassium disturbances.

CMT plays an important role in myocardial fibrosis development. Fibroblasts transform into cells with secretory and contractile phenotypes called myofibroblasts (Liu et al., 2021). Myofibroblasts do not exist in normal myocardial tissues, and quiescent fibroblasts can only transition into myofibroblasts under pathological conditions, thereby promoting collagen synthesis during myocardial fibrosis. In heart failure, various pathological stimuli that lead to heart damage, such as mechanical stress, metabolic disorders, or inflammatory stimuli, may activate myofibroblasts (Chen C. et al., 2016). During myocardial fibrosis, myofibroblasts are activated through the synergistic action of growth factors and matrix proteins, which transmit pro-fibrotic signals into the cell and promote α-SMA transcription and translation (Shinde and Frangogiannis, 2017). FAP, a membrane-bound proline-specific serine protease, is not expressed in normal fibroblasts but is expressed in myofibroblasts. Myofibroblasts express α-SMA and FAP, whereas fibroblasts do not. Therefore, α-SMA and FAP can be used as myofibroblast markers. Ang II, aldosterone, and TGF-β1 can promote the transformation and proliferation of myofibroblasts, which are therapeutic targets for the treatment of myocardial fibrosis (Shinde and Frangogiannis, 2014).

The specific molecular mechanisms of myocardial fibrosis are very complex and involve a series of intracellular signaling pathways. TGF-β1 is a major cytokine that promotes fibrosis in the DCM. Ang II stimulates the expression of TGF-β1 in cardiac fibroblasts, and locally produced Ang II can exert a powerful stimulating effect on cardiac fibroblasts directly or through TGF-β1-mediated pathways (Kagami et al., 1994; Massagué, 2012). TGF-β1 may be the downstream molecule that MR induces and promotes fibrosis. Aldosterone stimulates TGF-β1 expression in rat mesangial cells by enhancing ERK1/2 and JNK activities and subsequent AP-1 activity, promoting proliferation and fibrosis in inflammatory kidney disease (Han et al., 2009). In this study, we found that a high-glucose environment substantially increased the expression of TGF-β1; after knocking down ADAM17, the shearing of ACE2 decreased, and the TGF-β1 pathway was inhibited to reduce the degree of myocardial fibrosis. In addition, eplerenone competes with aldosterone for the mineralocorticoid receptor, inhibited the effect of aldosterone, reduced TGF-β1 expression, and reduced the degree of fibrosis. Finally, the combination of ADAM17 knockdown and eplerenone treatment reduced the level of TGF-β1 expression and further reduced the degree of fibrosis compared with that achieved with single therapy alone.

Numerous studies have shown that RAAS members, especially Ang II and aldosterone, play a key role in the pathogenesis of myocardial fibrosis. In a number of large-scale randomized clinical trials, ACEIs, ARBs and aldosterone receptor antagonists have significantly reduced mortality in patients with chronic heart failure, partly as a result of improved myocardial fibrosis and left ventricular remodeling. By blocking MR, eplerenone may attenuate cardiac steatosis and apoptosis and the subsequent remodeling and diastolic dysfunction in obese/type-II diabetic rats without inhibiting the effect of Ang II (Ramirez et al., 2013). The combination of ACEIs or ARBs and aldosterone receptor antagonists may benefit significantly in patients with heart failure who have normal renal function and potassium levels. However, the combination of ACEIs or ARBs and aldosterone receptor antagonists may cause severe hyperkalemia and thus have limitations. Our previous study found that ADAM17 knockdown mitigates while ADAM17 overexpression aggravates cardiac fibrosis and dysfunction, but it is unclear whether ADAM17 inhibition in combination with eplerenone treatment is better than single therapy and has no side effects. Our study found that ADAM17 inhibition combined with eplerenone significantly improved myocardial fibrosis, apoptosis, and inflammation in diabetic mice by dual inhibition of Ang II and aldosterone, without affecting blood potassium concentration. In addition, due to the presence of intracellular RAAS in the heart, currently available RAAS inhibitors may not provide the expected cardiovascular benefits in diabetic cardiomyopathy. An interesting approach is to exploit the protective facet of RAAS. This alternative prong of RAAS constitutes of ACE2, which cleaves angiotensin II to form angiotensin (1–7), promotes the antifibrotic effects of DCM through AT2R and MasR, and ADAM17 knockdown plays a protective role in DCM by increasing ACE2 expression. Several small molecule inhibitors of ADAM17 have been screened for cancer research, which provides conditions for the future development of ADAM17 small molecule inhibitors for diabetic cardiomyopathy, with great clinical translational potential. Taken together, our findings highlight the potential translational value of ADAM17 inhibition in combination with eplerenone after diabetic cardiomyopathy.

## Conclusion

In summary, ADAM17 knockout reduced diabetes-induced collagen synthesis and ameliorated cardiac remodeling through the inhibition of RAAS overactivation when combined with eplerenone treatment, which reducing TGF-β1/Smad3 pathway activation-mediated CMT. The combined intervention of ADAM17 knockout and eplerenone therapy provided additional cardiac protection compared with a single therapy alone, and did not disturb potassium level. Therefore, this is be a novel and promising finding for the treatment of DCM.

## Data Availability

The original contributions presented in the study are included in the article/Supplementary Material, further inquiries can be directed to the corresponding authors.
