# Supplementary material for: Combination of ADAM17 knockdown with eplerenone is more effective than single therapy in ameliorating diabetic cardiomyopathy
Source: Front Pharmacol. 2024 May 10;15:1364827. doi: 10.3389/fphar.2024.1364827 (PMC11122002; doi:10.3389/fphar.2024.1364827)
Supplement: Supplementary file 1 [file DataSheet1.zip › Supplementary Meterials.docx]

Supplementary Materials for

**Combination of ADAM17 knockdown with eplerenone is more effective than single therapy in ameliorating diabetic cardiomyopathy**

Lin Xie^1^, Dejin Zang^1^, Jianmin Yang^1^, Fei Xue^1*^, Wenhai Sui^1*^, Yun Zhang^1,2*^

Correspondence to: Yun Zhang, Email: zhangyun@sdu.edu.cn, or Wenhai Sui, E-mail: swh@email.sdu.edu.cn, or Fei Xue, E-mail: xuefei93xf@163.com.

This file includes:

Supplementary Figure S1 to S3

Supplementary Table 1 to 4


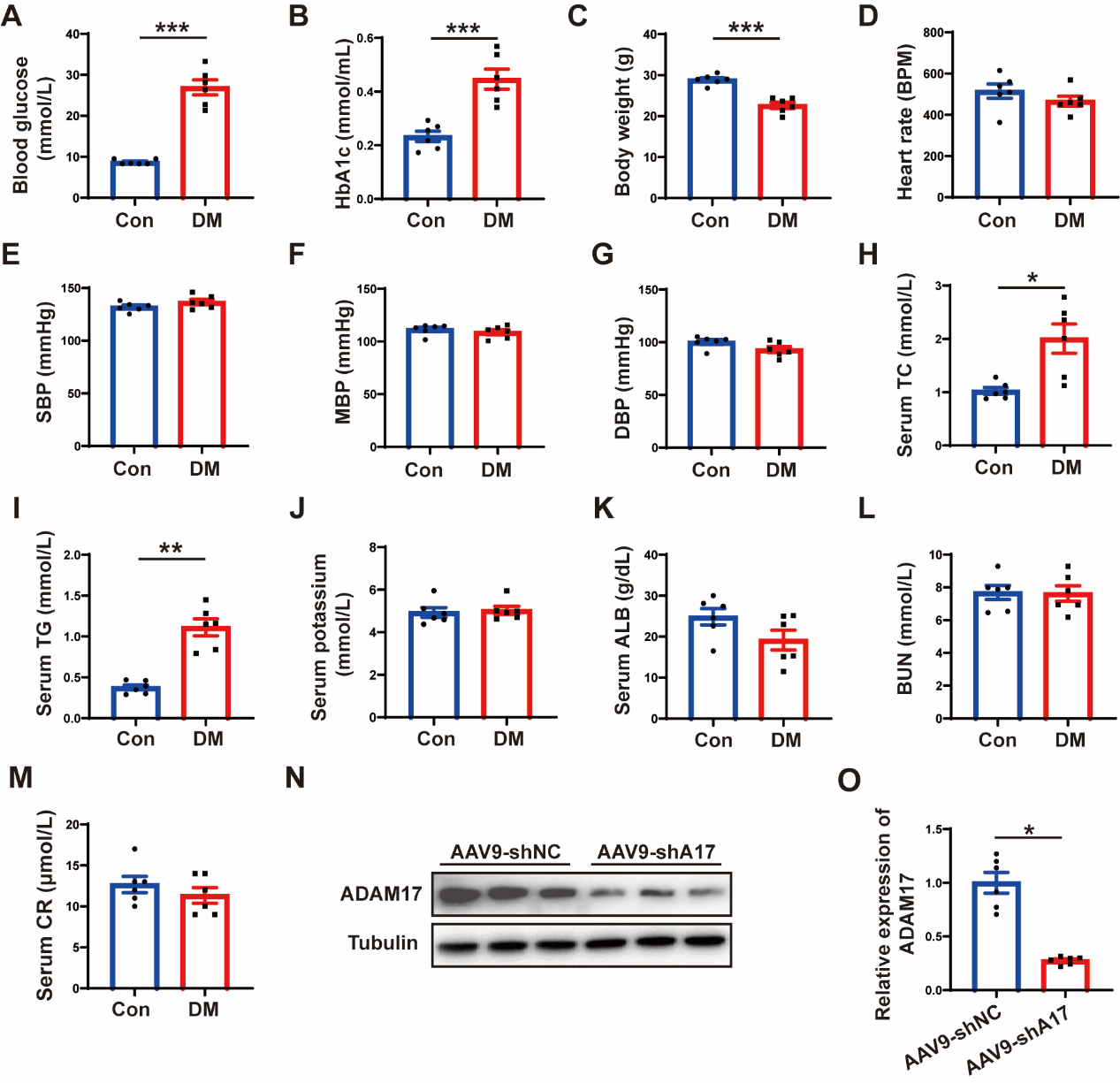


**Supplementary Figure 1. Basic characteristic of control and diabetic mice and the efficiency of ADAM17 knockdown in mice. A.** The blood glucose in two groups of mice. **B.** The serum Hemoglobin A1c (HbA1c) in two groups of mice. **C.** The body weight in two groups of mice. **D.** The heart rate in two groups of mice. **E.** The systolic blood pressure (SBP) in two groups of mice. **F.** The mean blood pressure (MBP) in two groups of mice. **G.** The diastolic blood pressure (DBP) in two groups of mice. **H.** The serum total cholesterol (TC) in two groups of mice. **I.** The serum triglyceride (TG) in two groups of mice. **J.** The serum potassium in two groups of mice. **K.** The serum albumin (ALB) in two groups of mice. **L.** The blood urea nitrogen (BUN) in two groups of mice. **M.** The serum creatinine (CR) in two groups of mice. **N-O.** The representative Western blot images and quantification analysis of ADAM17 protein expression in AAV9-shNC mice and AAV9-shA17 mice. Values shown were mean and SEM (n=6 in each group). **p* < 0.05; ***p* < 0.01; ****p* < 0.001.


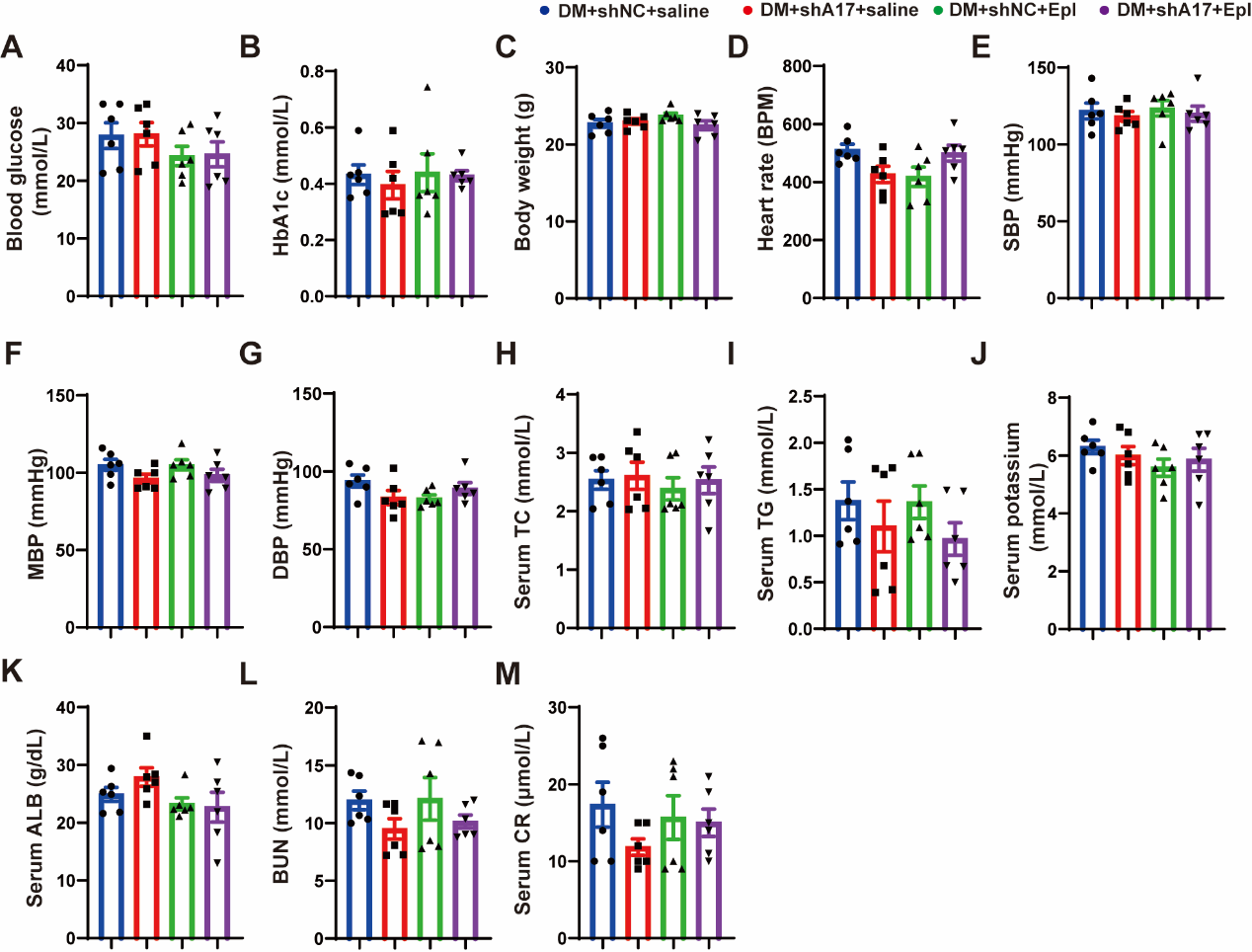


**Supplementary Figure 2. Basic characteristic in four groups of mice. A.** The blood glucose in four groups of mice. **B.** The serum HbA1c in four groups of mice. **C.** The body weight in four groups of mice. **D.** The heart rate in four groups of mice. **E.** The systolic blood pressure (SBP) in four groups of mice. **F.** The mean blood pressure (MBP) in four groups of mice. **G.** The diastolic blood pressure (DBP) in four groups of mice. **H.** The serum total cholesterol (TC) in four groups of mice. **I.** The serum triglyceride (TG) in four groups of mice. **J.** The serum potassium in four groups of mice. **K.** The serum albumin (ALB) in four groups of mice. **L.** The blood urea nitrogen (BUN) in four groups of mice. **M.** The serum creatinine (CR) in four groups of mice. Values shown were mean and SEM (n=6 in each group).


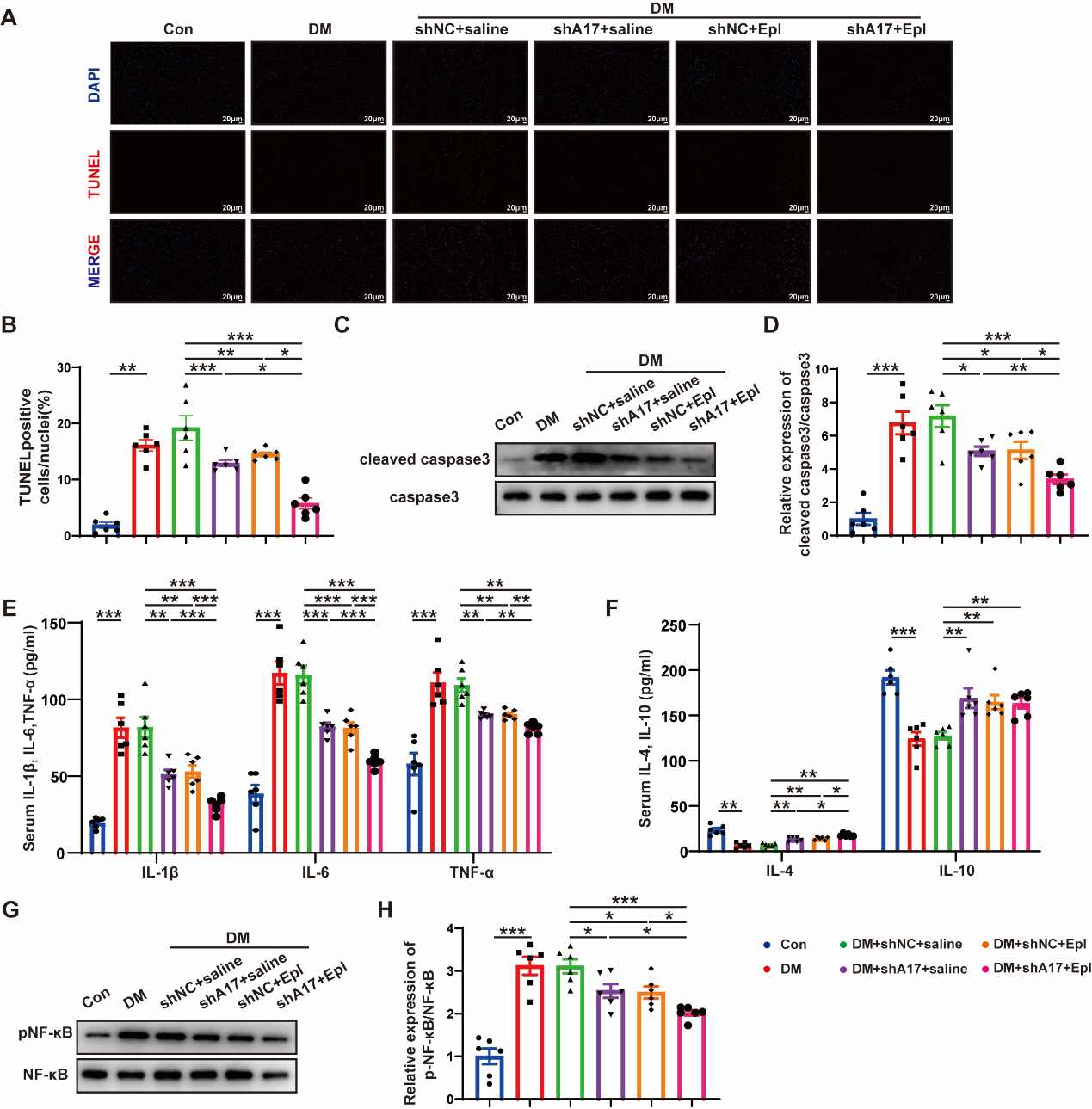


**Supplementary Figure S3. Cardiac apoptosis and inflammation following ADAM17 knockdown, eplerenone intervention and combined-administration in six groups of mice. A.** Representative TUNEL staining in six groups of mice (scale bar=20μm). **B.** Comparison of TUNEL-positive cells in the myocardium among six groups of mice. **C.** Representative Western blotting images of caspase 3 and cleaved caspase 3 expression in the myocardium of six groups of mice. **D.** Comparison of cleaved caspase3/caspase3 expression among six groups of mice. **E.** Serum levels of IL-1β, IL-6 and TNF-α in six groups of mice. **F.** Serum levels of IL-4 and IL-10 in six groups of mice. **G.** Representative Western blotting images of NF-κB and phospho-NF-κB expression in the myocardium of six groups of mice. **H.** Comparison of pNF-κB/NF-κB expression among six groups of mice. Values shown were mean and SEM (n=6 in each group). **p* < 0.05; ***p* < 0.01; ****p* < 0.001.

**Supplemental Table 1. Animal model and grouping**

| Groups | Part I | | Part II | | | | | | |
| --- | --- | --- | --- | --- | --- | --- | --- | --- | --- |
|  | Con  (n=6) | DM  (n=6) | DM+shNC+saline  (n=6) | | | DM+shA17+saline  (n=6) | | DM+shNC+Epl (n=6) | DM+shA17+Epl (n=6) |
| Week 0 | Vehicle injection for 5 days | STZ injection for 5 days | | STZ injection for 5 days | | | | | |
| Week 12 | Euthanasia | | AAV9-shNC  tail vein injection | | AAV9-shA17  tail vein injection | | AAV9-shNC  tail vein injection | | AAV9-shA17  tail vein injection |
| Week 14 |  |  | saline administration | | | saline administration | | Epl administration | Epl administration |
| Week 18 |  |  | Euthanasia | | | | | | |

Con, control; DM, diabetes mellitus; STZ, streptozotocin; shNC, negative control AAV9-shRNA; shA17, ADAM17 AAV9-shRNA; Epl, eplerenone.

**Supplemental Table 2.** **Cell grouping and treatment**

| Groups | LG  (n=6) | HG (n=6) | HG+siNC+saline  (n=6) | HG+siA17+saline (n=6) | HG+siNC+Epl  (n=6) | HG+siA17+Epl (n=6) |
| --- | --- | --- | --- | --- | --- | --- |
| Hour 0 |  |  | NC-siRNA transfection | A17-siRNA transfection | NC-siRNA transfection | A17-siRNA transfection |
| Hour 24 |  |  | saline treatment | | Epl treatment | |
| Hour 48 | 5.5 mM glucose treatment | 60 mM glucose treatment | | | | |
| Hour 72 | cell collection | | | | | |

LG, low glucose; HG, high glucose; siNC, negative control-siRNA; siA17, ADAM17-siRNA; Epl, eplerenone.

**Supplemental Table 3.**

| **Antibodies** | **Source** | **Identifier** |
| --- | --- | --- |
| ADAM17 WB: 1:1000 | Abcam | Cat# ab2051 |
| Collagen III  WB: 1:1000  IHC: 1:100 | Abcam | Cat# ab7778 |
| Collagen I  WB: 1:1000  IHC: 1:100 | Abcam | Cat# ab34170 |
| tubulin WB: 1:1000 | Abcam | Cat# ab6046 |
| FAP  WB: 1:1000  IHC: 1:100 | Abcam | Cat# ab314456 |
| α-SMA WB: 1:1000 | Cell Signaling Technology | Cat# 19245 |
| TGF-β1 WB: 1:1000 | Abcam | Cat# ab215715 |
| ACE2 WB: 1:1000 | Abcam | Cat# ab108252 |
| AT1R WB: 1:1000 | Abcam | Cat# ab124734 |
| AT2R WB: 1:1000 | Abcam | Cat# ab92445 |
| Smad3 WB: 1:1000 | Cell Signaling Technology | Cat# 9523 |
| Phospho-Smad3  WB: 1:1000 | Cell Signaling Technology | Cat# 9520 |
| GAPDH WB: 1:1000 | Cell Signaling Technology | Cat# 5174 |
| Caspase3 WB: 1:1000 | Cell Signaling Technology | Cat# 9662 |
| Cleaved caspase3  WB: 1:1000 | Cell Signaling Technology | Cat# 9664 |
| NF-κB WB: 1:1000 | Cell Signaling Technology | Cat# 8242 |
| Phospho-NF-κB  WB: 1:1000 | Cell Signaling Technology | Cat# 3033 |

**Supplemental Table 4. Primers used for RT-PCR analysis.**

| Gene | Sequence 5’-3’ | Primers |
| --- | --- | --- |
| *β-mhc* (Mus) | GCTCAGCAATCTATTTGCCAAC | Forward |
|  | AGCCTTTCTTTGCCTTGCCT | Reverse |
| *β-actin* (Mus) | CACTGTGCCCATCTACGA | Forward |
|  | GTAGTCTGTCAGGTCCCG | Reverse |
